# Beyond Jump Height: A Comparison of Concentric Variables in the Squat Jump, Countermovement Jump and Drop Jump for Athletic Profiling

**DOI:** 10.3390/sports13110379

**Published:** 2025-11-03

**Authors:** Hamish Kyne, John B. Cronin

**Affiliations:** Sports Performance Research Institute New Zealand, Health and Environmental Sciences, Auckland University of Technology, Rosedale, Auckland 0632, New Zealand

**Keywords:** countermovement jump, drop jump, squat jump, stretch-shortening cycle, vertical jump

## Abstract

Jump height provides limited insight into movement strategies and muscle-tendon unit (MTU) contraction dynamics of the squat jump (SJ), countermovement jump (CMJ), and drop jump (DJ). This study investigated whether concentric phase variables differ significantly across these jumps. Twenty-three male athletes (age: 18.1 ± 0.7 years) from various sports performed the SJ, CMJ and DJ on a force plate. Variables included jump height (JH), concentric duration (ConT), concentric mean force (CMF), relative concentric mean force (rCMF), and concentric force index (CFI). Significant differences (*p* < 0.05) were observed in all variables across jump types. The CMJ led to the greatest JH (37.8 ± 4.1 cm) compared to SJ (34.6 ± 4.4 cm) and DJ (31.9 ± 5.0 cm). Conversely, the DJ produced the greatest rCMF (30.1 ± 2.7 N/kg) within the shortest ConT (125 ± 15 ms), compared to the SJ (16.2 ± 0.8 N/kg in 409 ± 48 ms) and CMJ (19.8 ± 1.0 N/kg in 273 ± 24 ms). Minimal shared variance and varied individual athlete rankings across jumps suggest that each jump elucidates different facets of MTU function. As a novel variable, the CFI showed poor correlation with JH (r = −0.103 to 0.002), suggesting it may provide insights beyond jump height alone. These findings support our hypothesis that SJ, CMJ, and DJ offer distinct diagnostic insights into varying MTU contraction dynamics and the physical components underpinning jump performance, indicating that longitudinal monitoring of the CFI and underlying components (rCMF/ConT) across these jumps could enhance athletic profiling.

## 1. Introduction

When a concentric muscle contraction is preceded by an eccentric muscle contraction, concentric force, velocity, and power output is augmented [1,2,3,4]. This phenomenon is termed the stretch-shortening cycle (SSC). The effectiveness of the SSC is reliant on the structural properties of the muscle-tendon unit (MTU) and neurological factors.

To explore how these structural properties are involved in mechanical force transmission during the SSC, the three-component model (TCM) originally conceptualized by Hill [5,6,7] is commonly used. This model delineates the roles of the contractile component (CC), series-elastic component (SEC), and parallel elastic component (PEC). The CC represents the muscle’s active force-generating mechanisms, described by the force–velocity and force–length relationships [5], whereas the SEC and PEC consist of distinct categories of anatomically distributed elastic structures based on their positioning relative to the CC. In particular, the SEC transmits active force generated by the CC to skeletal structures through elastic elements (primarily tendons and titin) arranged in series with the CC [6,8]. The PEC consists of intramuscular connective tissues in parallel with the CC, including the endomysium, perimysium, and epimysium [9,10]. These tissues provide passive elasticity to the MTU; however, at shorter than resting muscle lengths, the PEC does not contribute to force transmission. At greater than resting muscle lengths force is the sum of the PEC and titin passive resistance and active force generated by the CC [11]. Although the CC, SEC, and PEC cannot be isolated, their contribution and interaction are influenced by the specific characteristics of athletic movement [12,13,14,15].

Researchers using in vivo investigations have shown that contraction type, movement velocity, movement amplitude, and which joints are involved all contribute to the various lengthening and shortening of the CC, SEC, and PEC in dynamic movements [12,16,17,18,19,20,21,22,23,24,25]. Vertical jumps have been traditionally utilized as a practical method to indirectly assess the function of the SSC, as they are simple and efficient to perform [26,27]. The most frequently utilized vertical jump is the countermovement jump (CMJ) [26,27,28,29,30,31,32], followed by the squat jump (SJ) and drop jump (DJ). These three jump variations have various contraction types, movement velocities, amplitudes, and joint involvement [13,33,34,35]; therefore, they could theoretically provide unique diagnostic insight into the various contributions of contractile and elastic components.

The SJ is characterized by a pause for 3–4 s following an athlete’s descent into the squat position before executing the jump; the stored elastic energy is dissipated as heat [36]. As no eccentric contraction precedes the jump, the SJ serves as a baseline measure of an athlete’s capacity to concentrically produce force, which reflects the force largely produced by the CC. In contrast, the CMJ is characterized by its countermovement with a total contraction time varying from 350 to 1300 ms [37,38,39], which is commonly classified as a slow SSC movement [40]. The CMJ’s slow contraction velocity and relatively large movement amplitude suggests not only the involvement of the CC and SEC but also a significant role of the PEC, especially when considering the lengthening of the CC beyond its resting length during the eccentric phase [17]. Further, the DJ involves an external preload by dropping from a predetermined height prior to the jump. Although DJ ground contact times (GCT) vary according to drop height and instructions, it has been shown that with correct technique and cueing, GCT should not exceed 250 ms and therefore is often classified as a fast SSC movement [41,42]. This jump variation is executed with smaller angles in the hip and knee joints than the SJ and CMJ [41]; the quick GCT and small movement amplitudes indicate minimal involvement of the PEC, and any elastic enhancement indicates a function of SEC contribution [41,42].

The inherent differences between these three jumps, when viewed through the lens of the TCM, suggest that they could provide valuable insight into various qualities and structural aspects of the SSC. This is not to say that these jumps isolate each component, but rather the contribution of each component changes in magnitude depending on amplitude and velocity of movement. The majority of research investigating these jumps has done so mainly in isolation, bilaterally, or with an outcome metric such as jump height (JH), which may be limiting. As an outcome variable, JH may not always be the desired adaptation and/or does not provide insight into the movement strategies that produce the outcome. Utilizing force plates to understand underlying movement strategy metrics may help to enhance athletic performance. Further, the CMJ and DJ are often compared utilizing metrics such as the reactive strength index (RSI) or reactive strength index modified (RSImod) and/or investigating variables of interest from the eccentric phase. As the SJ is exclusively concentric, the comparison of common metrics across these three jump types could provide interesting insights into movement strategy and tissue contribution; therefore, concentric variables are the focus of this study.

To the best of the author’s knowledge, no researchers have focused on comparing the concentric variables of these jumps. With the proliferation of force plates, there has been an ever-increasing number of metrics to select from [43,44] that is overwhelming for many practitioners. Simply, the goal of physiotherapists is to restore force capability in the injured, and for the strength and conditioning coach to maintain or increase force production, decrease the time required to produce force, or both in the athlete. Consequently, comprehending an athlete’s capacity to generate force, the duration over which they can produce this force, and the variations in these parameters among the SJ, CMJ, and DJ could yield invaluable diagnostic information. As a novel metric, the concentric force index (CFI) is introduced and provides a novel perspective for comparing these jumps. By quantifying the mean force exerted during the concentric phase relative to its duration, the CFI provides a standardized metric to contrast the three jumps, potentially revealing differences in MTU contraction dynamics amongst athletes. As such, the aim of the study was to compare concentric SJ, CMJ, and DJ variables to determine if they differ significantly and to explore whether such differences may reflect varying contributions of the CC, SEC, and PEC. It is hypothesized that (1) concentric variables of interest will significantly differ due to the unique biomechanical demands of each jump; (2) despite shared underlying athletic abilities, the shared variance between variables will be limited, suggesting that the jumps are more dependent on different parts of the TCM; and (3) due to these differences, individual participant rankings will vary across concentric variables, indicating potential areas for targeted training to enhance specific MTU components.

## 2. Materials and Methods

### 2.1. Participants

Twenty-three male athletes (age: 18.1 ± 0.7 years; weight: 77.9 ± 8.3 kg; height: 182.9 ± 5 cm) volunteered to participate in this study. The cohort included athletes from a diverse range of sports, including rugby, football, cricket, hockey, tennis, basketball, and athletics. All participants were free of medical issues or injuries that could compromise performance and had at least 2 years of structured training in their primary sport. Subjects were informed of the protocol and procedures prior to their involvement, and written consent to participate was obtained. The Institutional Ethics Committee of Auckland University of Technology provided approval for this study. This research was approved by the Auckland University of Technology Ethics Committee (AUTEC 20/105). A post hoc power analysis (G*Power 3.1.9.7) confirmed achieved power = 1.00 for the repeated-measures ANOVA (effect size f = 1.15–9.95, derived from partial η^2^ = 0.57–0.98; α = 0.05, ρ = 0.2, ε = 1), indicating the sample size was sufficient to detect the large effects observed.

### 2.2. Procedures

This study employed a repeated-measures design to compare concentric phase variables across the SJ, CMJ, and DJ in a single testing session. Subjects were required to avoid stressful physical activity 24 h beforehand. Prior to testing, subject information [age, body mass (scales), and height (stadiometer)] was collected. Subjects were informed of the procedures and, following a 10 min standardized warm-up, were familiarized with each vertical jump test until technique was verified as correct by the researcher. Subjects then performed either the SJ, CMJ, or DJ trials in a randomized order to minimize any order or fatigue effects. The subjects were required to maximally attempt each jump for three consecutive trials. Therefore, each subject was required to complete a minimum of nine jumps (3 jump conditions × 3 jumps). Each consecutive trial was separated by at least 30 s and each jump condition by at least a 2 min rest period [45,46]. The best two performances were averaged and used for analysis.

### 2.3. Vertical Jump Testing

To improve the reliability of the data, participants were instructed to jump with their knees and ankles extended and land in a similarly extended position [47]. To limit the impact of instructions on performances, consistent instructions were provided to all participants during each vertical jump trial [48]. All participants were instructed to “jump as high and fast as possible off the force plate” for each jump variation. All jumps were performed with hands on hips to prevent the use of arms and attain true measures of leg force–time variables [48]. If the subject removed their hands from hips, displayed excessive knee flexion whilst airborne, or landed in an incorrect position, the jump was declared invalid and repeated after adequate rest [49].

To perform the squat jump, subjects started in a standing position, with feet hip width apart on the force plate. Prior to jumping, the participant stood still for 2–3 s to determine system weight which was used for analysis. Participants were instructed to lower themselves into a squat to approximately a 90° knee angle (as determined by the investigator), hold for four seconds [36], then jump as high as possible, attempting to eliminate any countermovement. If a countermovement was observed the SJ was repeated. To perform the CMJ the subjects began in a standing position, with feet hip width apart on the force plate. Prior to jumping, the participant stood still for 2–3 s to determine system weight, which was used for subsequent analysis. Subjects initiated the jump with a countermovement consisting approximately of a 90° knee angle (as determined by the investigator), instantly followed by a maximal effort vertical jump. Participants were instructed to jump for maximum height and ensure there was no pause between the eccentric and concentric phases. The DJ was performed from a standardized height of 30 cm [50]. During the DJ, subjects were instructed to step out and fall onto the force platform. Further instructions to minimize GCT included to “jump as fast and as high as possible off the force plate”. After the second landing, participants were required to stand still for 2–3 s to attain the system weight which was used for analysis.

### 2.4. Data Collection

All vertical jump data was collected with a portable force plate (AMTI, ACP, Watertown, MA, USA) using a sampling rate of 1000 Hz. Raw vertical ground reaction force (vGRF) data was analyzed using ForceDecks software V2.0.9064 (VALD, Brisbane, Australia) to calculate the variables of interest. The initiation of the concentric phase of the SJ was when vGRF exceeded 20 N above system weight [51]. Initiation of the CMJ was defined as the point when total vGRF deviated −20 N from system weight. Initiation of the landing of the DJ was when vGRF exceeded 20 N. All take-offs were identified as the instant when vGRF fell below 10 N. The impulse–momentum relationship was used to calculate JH. Where appropriate, contraction time was divided into an eccentric phase and a concentric phase. The start of the concentric phase was determined when velocity of the centre of mass became positive. The concentric force index (CFI) was calculated as concentric mean force relative to bodyweight (CMF/kg) divided by concentric duration (ms).

### 2.5. Statistical Analysis

Means and standard deviations were used as measures of centrality and spread of data. Repeated-measures ANOVA with Bonferroni post hoc contrast was utilized to determine if the measures of interest were significantly different between the three jump conditions. Correlations between variables were assessed with Pearson’s correlation coefficients. Correlations were interpreted as trivial (0.0–0.1), small (0.1–0.3), moderate (0.3–0.5), large (0.5–0.7), very large (0.7–0.9), and nearly perfect (0.9–1.0). Further, R^2^ values were reported to investigate the shared variance of these variables. The level of significance was set at *p* < 0.05 for all statistical tests. SPSS 29.0 (SPSS Inc., Chicago, IL, USA) was used for statistical analysis.

## 3. Results

Means and standard deviations are presented in Table 1. Significant differences were observed between all variables of interest. Notably, the CMJ produced significantly greater JH compared to both SJ (8.9%, *p* < 0.001) and DJ (16.8%, *p* < 0.001), whereas there was no significant difference between the SJ JH and DJ JH. Furthermore, significant differences in ConT, CMF, and rCMF among all jump types were observed (*p* < 0.001).

The correlations among variables across the different jump types are presented in Table 2. When investigating JH between jump types, a large positive relationship between the SJ and CMJ (r = 0.69, *p* < 0.001) with a shared variance of 47.6% was observed. Conversely, a moderate negative correlation emerged between CMJ and DJ ConT (r = −0.45, *p* < 0.05). Regarding CMF, correlations between jumps varied from large to very large (r = 0.54–0.9, *p* < 0.05) with a shared variance of 29.4–81.2%. However, no significant relationship was observed for rCMF whilst R^2^ remained below 9.3% for all three comparisons. The relationships between JH and other variables of interest for each jump type are presented in Table 3. No significant relationship was observed between JH and any other variable for any of the jumps.

**Figure 1:**
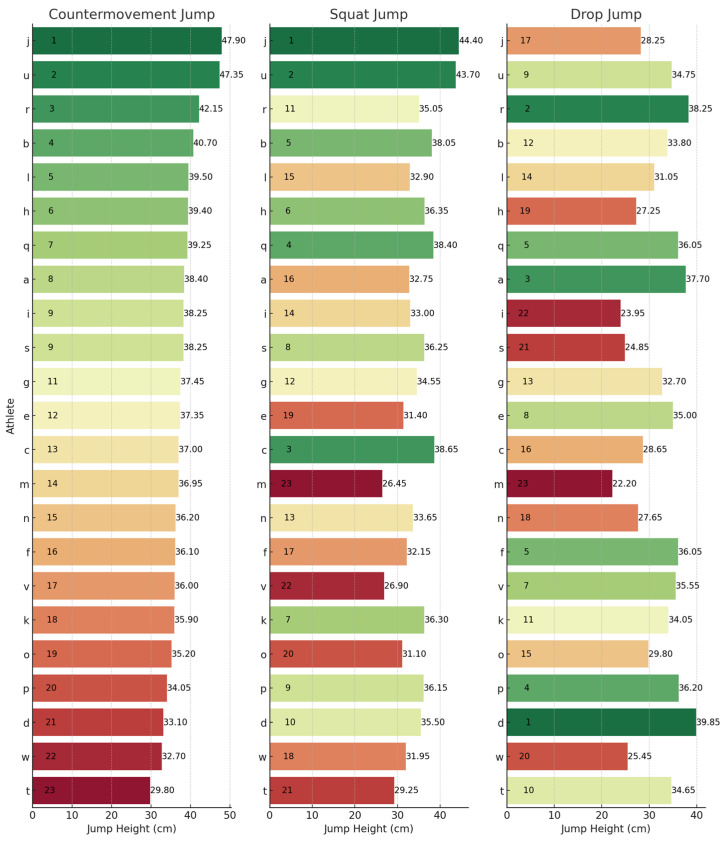
Jump height rankings across the CMJ, SJ, and DJ. Letters (a–w) represent anonymized athlete identifiers. Numbers indicate rankings (1 = best performance) or specific jump height values (cm). Colours represent a performance gradient: green for best, transitioning to red for worst.

**Figure 2:**
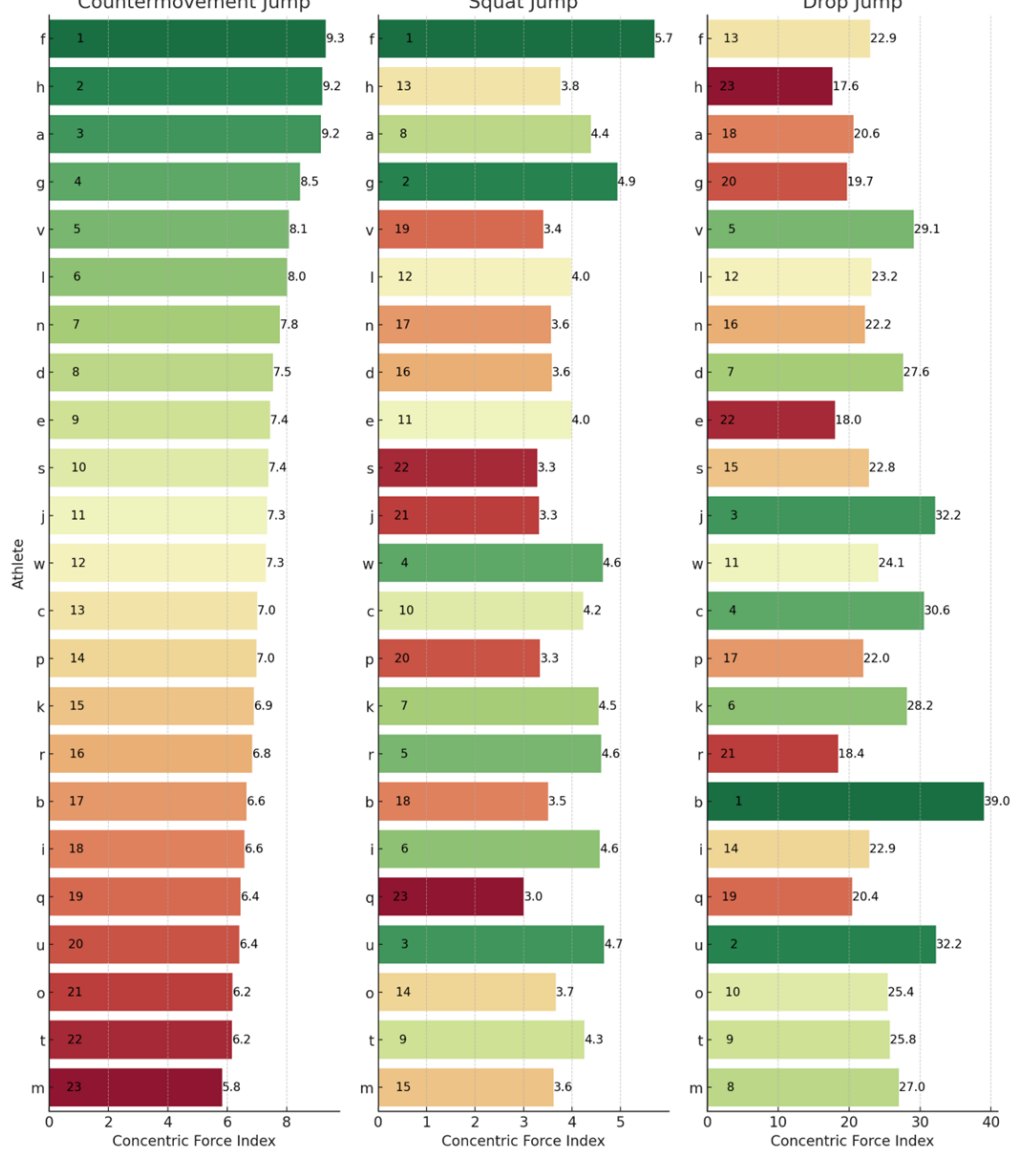
Concentric force index rankings across the CMJ, SJ, and DJ. Letters (a–w) represent anonymized athlete identifiers. Numbers indicate rankings (1 = best performance) or specific concentric force index values. Colours represent a performance gradient: green for best, transitioning to red for worst.

**Figure 3:**
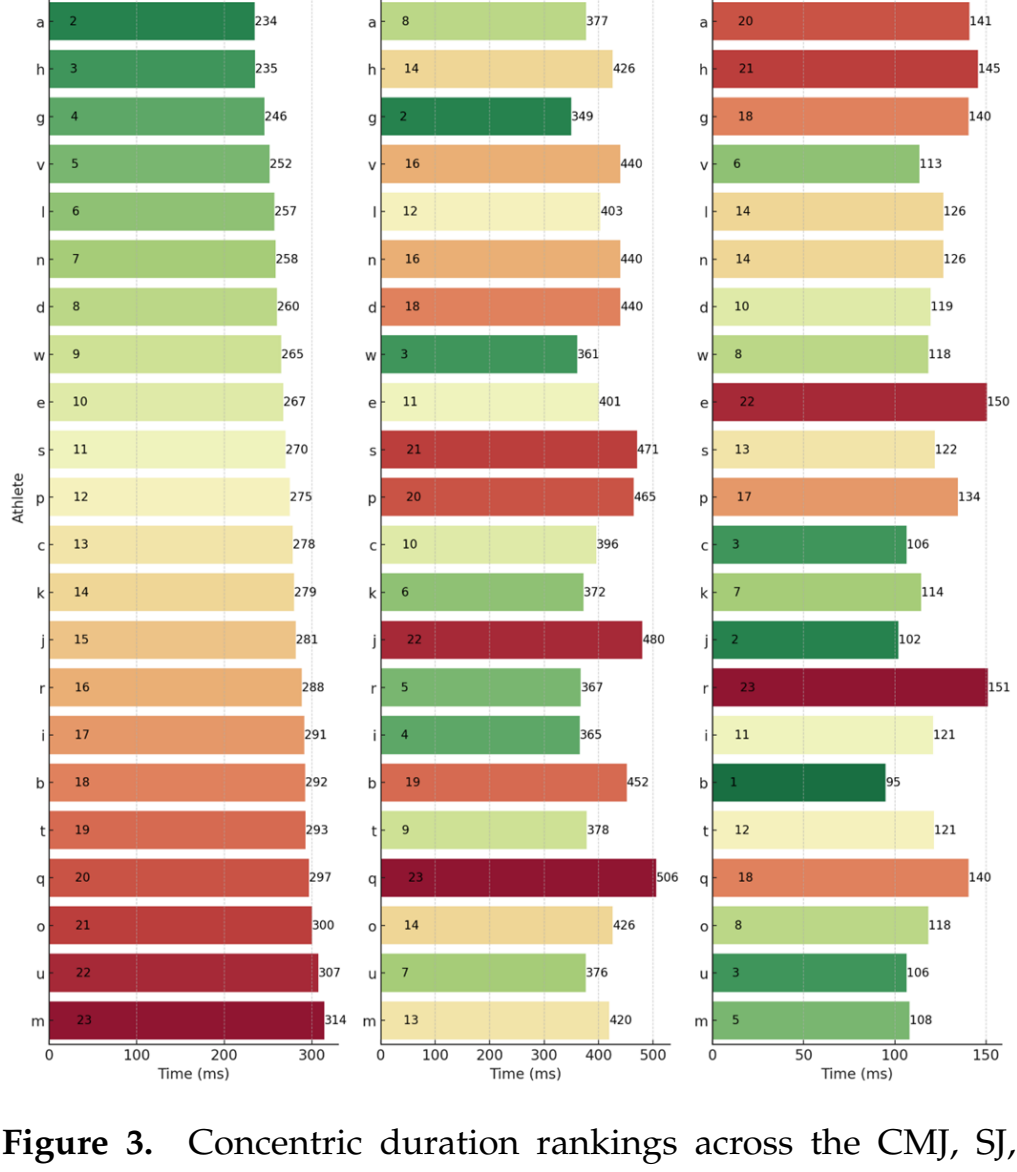
Concentric duration rankings across the CMJ, SJ, and DJ. Letters (a–w) represent anonymized athlete identifiers. Numbers indicate rankings (1 = best performance) or specific concentric duration values (ms). Colours represent a performance gradient: green for best, transitioning to red for worst.

**Figure 4:**
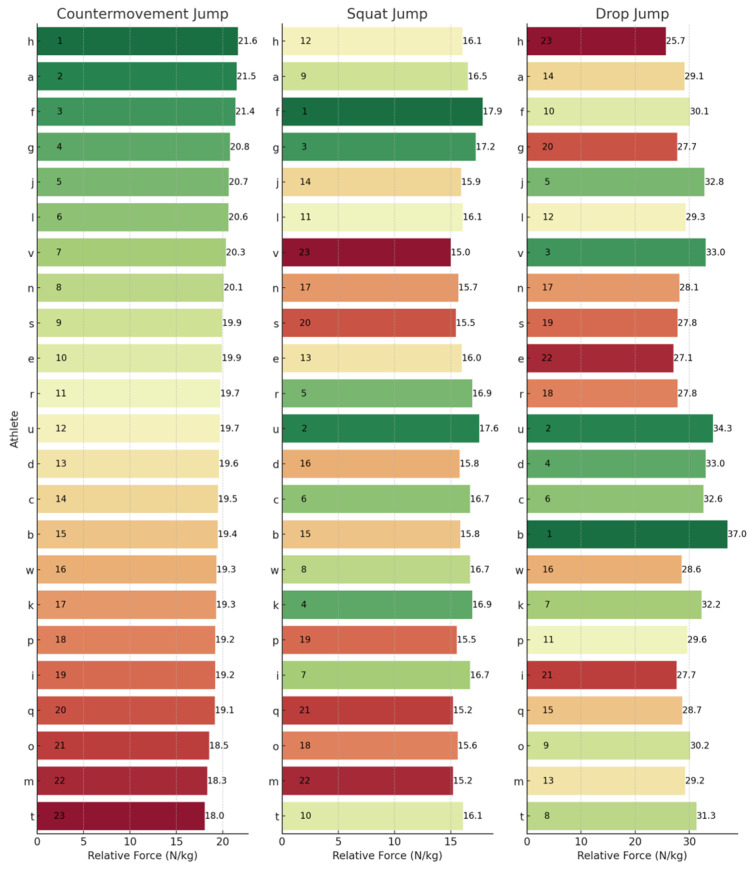
Relative concentric mean force rankings across the CMJ, SJ, and DJ. Letters (a–w) represent anonymized athlete identifiers. Numbers indicate rankings (1 = best performance) or specific relative concentric mean force values (N/kg). Colours represent a performance gradient: green for best, transitioning to red for worst.

To provide a granular perspective on individual performances across the different metrics, athlete rankings for each jump type have been illustrated. These can be observed below for JH (Figure 1), CFI (Figure 2), ConT (Figure 3), and rCMF (Figure 4). Visually, it was observed that performance in the three jumps differed amongst the variables of interest. Notably, there was a discernible trend where athletes excelling in the CMJ also tended to rank highly in the SJ, particularly for JH. However, this trend was not observed in relation to the DJ. For example, athlete J had the highest JH in the CMJ (47.90 cm) and SJ (44.40 cm) whilst he was ranked 17th for the DJ (28.25 cm). In contrast, athlete D had the highest JH in the DJ of 39.85 cm and was ranked 21st and 10th for the CMJ (33.10 cm) and SJ (35.50 cm), respectively.

## 4. Discussion

The aim of this study was to investigate the diagnostic value of the SJ, CMJ, and DJ and whether these jumps provide distinct insights into athletic performance due to their varying MTU contraction dynamics. Notable key findings include the following: (1) the three jump types were all found to differ significantly in their JHs and concentric force profiles; (2) there was minimal shared variance between jump strategy metrics of the three jump types; (3) individual athletes exhibited variations in their rankings for each jump type, underscoring the unique demands associated with each movement; and (4) the CFI could differentiate between jumps and provide alternative insight to what JH can provide.

While this study interprets these findings primarily through the mechanical lens of lengthening and shortening of the TCM components (CC, SEC, PEC), the authors acknowledge that neural factors provide a complementary perspective that could also influence the observed concentric differences [52]. These neural variations may manifest mechanically as length changes in the CC, SEC, and PEC, while mechanical demands reciprocally shape neural strategies. Other contributing factors, such as reflex potentiation and intermuscular coordination, may also modulate these concentric profiles.

### 4.1. Comparison Between Jumps

The primary purpose of this study was to compare the JH and concentric force profiles between the SJ, CMJ, and DJ. It was observed that the CMJ led to significantly higher JH values than the SJ (9.2%) and DJ (18.5%). Previously, it has been observed that CMJ JH is typically 5–20% greater than SJ JH [1,2,4,37,39,47,53]. This is consistent with the observations of this study. Previously, when comparing DJ JH to the SJ or CMJ, researchers have not presented a unified view. In the current cohort, SJ JH was 8.5% greater than DJ JH, yet this difference was not statistically significant. Previously researchers have shown DJ JH to be greater than SJ JH [47,50,54] and either greater or equal to CMJ [47,55]; however, this was not observed in the current cohort. This discrepancy observed between findings may be influenced by various factors, including instructions given to participants. Participants in this study were instructed to minimize GCT to ensure they were performing a fast SSC DJ. This is in contrast to Earp et al. [50,54], where GCT was substantially longer (557 ± 117 ms). However, the absence of a clear hierarchy in JH among the three jump types may also imply that the athletes or cohorts possess different capabilities in utilizing the SSC or that the study designs had varying task requirements.

The concentric metrics for the SJ, CMJ, and DJ were also found to have large significant differences, highlighting the unique requirements for propulsive force production for each jump type. When compared to the SJ, rCMF was 22% greater in the CMJ and 86% greater in the DJ. Additionally, there were significant differences in ConT among the jumps, with the DJ exhibiting the shortest ConT (124 ms) and SJ displaying the longest (409 ms). This discrepancy was expected, given that the temporal requirements of the three jumps are influenced by range of motion. However, the significant differences between temporal variables in the concentric phase further reinforces that each of these jumps have different jump strategy requirements and, therefore, would likely require different MTU capabilities to deal with the unique biomechanical demands of each.

### 4.2. Shared Variance

Previously, shared variance has been utilized to discern whether various athletic tasks are measuring distinct motor qualities [56,57,58]. If the SJ, CMJ, and DJ all had a high degree of shared variance, it would be unlikely that they would provide any diagnostic utility. The greatest shared variance between jumps was observed in CMF (R^2^ = 29–81%). However, when evaluating the rCMF there were no significant relationships between jumps with shared variance ranging from 0 to 9%. This observation implies that the absolute force values may be conflated by the individual’s body mass, suggesting that relative force measures might provide a more precise understanding of an athlete’s force production capabilities independent of body weight.

Although the SJ and CMJ revealed a large positive relationship for JH (0.69, *p* < 0.001), and the shared variance between these jumps was 47.6%, DJ JH showed no substantial relationship with either SJ JH or CMJ JH. However, other than CMF across the jumps (R^2^ = 29.8%) and JH between SJ and CMJ (R^2^ = 47.6%), there were no other variables that had a shared variance greater than 20%. This provides evidence that each of these jumps assesses different qualities.

The moderate negative correlation between CMJ and DJ ConT (r = −0.452, *p* < 0.05) was unexpected. It appears that athletes that had shorter DJ ConT took longer in the concentric phase of the CMJ. Although this was unexpected, it is speculated that those that excelled in one jump had physical qualities that benefit performance in one jump over another. For example, an individual with longer limbs and greater elastic recoil may have a faster DJ concentric phase; however, these physical characteristics may also mean that their movement amplitude in the SJ and CMJ is larger and therefore increases ConT in the CMJ.

### 4.3. Rankings

The lack of substantial relationships and shared variance among the SJ, CMJ, and DJ underscores their assessment of distinct qualities, which are further emphasized by individual rankings in Figure 1, Figure 2, Figure 3 and Figure 4. For example, when observing JH, athletes J and U performed well in the SJ and CMJ but ranked poorly in DJ. Conversely, athlete D and P were both highly ranked in the DJ whilst ranking lower in the SJ and CMJ, indicating varying capabilities for individuals across jump types. Similar trends emerged with rCMF and ConT, with athlete H ranking highest for rCMF in CMJ but lowest in DJ, and athlete B excelling in DJ while ranking lower in SJ and CMJ. In fact, athlete B was able to produce 44% greater rCMF in the DJ than athlete H, highlighting the difference between an individual’s force-producing capabilities across these vertical jumps. Further, in Figure 3, the four athletes that had the fastest ConT in the CMJ were in the lowest quartile for the DJ, whereas three of the top five fastest in the DJ ConT were in the bottom quartile for the CMJ. These athletes were taking 38–53% longer during the concentric phase of the DJ when compared to the top ranked for DJ ConT.

It would seem that athletes in this cohort possessed varied capabilities and adopted distinct jump strategies accordingly. Theoretically, this may be due to either of the following; (a) athletes are more trained in a particular technique that is more beneficial to one particular jump; (b) athletes have particular anatomical advantages which are beneficial to particular jumps; or (c) parts of the TCM may be under-developed in certain athletes. For example, assuming technique and anatomical considerations remain constant, if an athlete improves their metrics in the CMJ and DJ without a corresponding improvement in the SJ, this may suggest an athlete could benefit from training focused on developing the CC. Furthermore, if an athlete already demonstrates sufficient active force production from the CC, monitoring the metrics with the goal of observing greater improvements in the DJ and CMJ may be advantageous, particularly for athletes who could benefit from greater elastic force augmentation.

### 4.4. Concentric Force Index

Recently, it has been suggested that outcome metrics such as JH provide a limited amount of information about jump performance [59]. The findings of this study support this claim, suggesting that JH may not be an optimal primary outcome variable to differentiate SSC qualities amongst these three jumps. JH performance only showed trivial to moderate correlations with key concentric metrics such as CMF and ConT across different jumps (Table 3), suggesting that there are multiple movement strategies to achieve a particular JH. These movement strategies are likely influenced by factors such as anatomy, body composition, and jumping technique [60].

Additionally, this limitation with JH has been observed in fatigue monitoring. Kennedy and Drake [61] observed that 24 h post-high-intensity training, CMJ JH, CMF, and ConT were impaired; however, at the 48 h mark, JH returned to baseline whereas CMF declined even further and ConT remained impaired. Although the athletes produced less force over a longer duration, the product was a similar JH to the pre-fatigued state. With less force and greater duration and fatigue present, theoretically there would likely be a change in the contributions of the CC, PEC, and SEC. Therefore, comparing JH without accounting for the underlying movement strategy metrics can be misleading.

In this study, SJ JH was greater than DJ JH on average; however, the ConT for the DJ was significantly shorter (124 ms compared to 410 ms for the SJ). Additionally, rCMF increased by 86% from 16.2 N/kg for the SJ to 30.1 N/kg for the DJ. This indicates that athletes were able to produce significantly more force over a much shorter interval during the DJ. These findings further highlight the importance of considering not only the jump height but also force production and temporal variables when assessing jump performance [62].

A current solution to account for movement strategy during the DJ and CMJ, which has gained considerable interest, is the RSI and RSImod [26,27,63]. Both metrics take into account JH and either GCT (RSI) or total contraction time (RSImod). However, as the DJ and CMJ both contain eccentric phases during ground contact time (GCT) and total contraction time (TCT), respectively, these values are not comparable to the total contraction time of the SJ. Further, although RSI and RSImod are both simple and highly reliable [63,64,65,66], both measures come with limitations. Firstly, they do not account for body mass. If an athlete has put on 10 kg and RSI has remained the same, this would suggest that the athlete’s force-producing capabilities have improved. Secondly, if RSI increases, is this due to a shorter GCT or a higher jump? Two athletes could attain the same RSI value, one who has developed a rapid force and another who has spent longer on the ground developing force. These are two completely different training adaptations that are not distinguishable from RSI alone. However, both scenarios have implications for the practitioner. Healy et al. [67] suggested that coaches should limit variability in jumping performance by implementing GCT/TCT thresholds. This would ensure that any changes to RSI would be due to JH and allow performances to be compared between individuals. However, in this situation, JH may as well be the outcome metric of choice whilst controlling for GCT/TCT. For these reasons, it has been suggested that RSI/RSImod should always be presented with the underlying JH and GCT/TCT to provide context [26,67,68].

By investigating the relative mean force exerted during the concentric phase relative to the duration of the concentric phase, the CFI provides a different viewpoint from that of JH and RSI/RSImod. For example, in this study, athletes J and U both exhibited low CFI scores relative to their JH performance. These athletes were both basketball players with an extensive background in jump training but little resistance training. The argument could be made that they had a relatively high JH compared to the rest of the group and therefore are the most physically prepared. However, an argument could be made for the opposite—that jump technique is masking physical capacity limitations. A low CFI score suggests that they were not producing much force and/or not producing it very quickly. Further, in sports where vertical JH is not pivotal, JH is often overly focused on. A paradigm shift is required where greater emphasis is placed on the underlying metrics that drive athleticism. Utilizing vertical jump tests on force plates provides the capability to assess how much force an athlete can produce and over what time period, both of which are critical components of athletic success. Optimizing force production for a particular jump over a particular duration may be of more benefit to an athlete than optimizing training to improve JH.

The CFI utilized in this study, however, is not immune to limitations. Firstly, as outlined previously, as it is a ratio, both rCMF and ConT would both need to be monitored. Another expected criticism of the CFI is that it does not provide any additional information than the RSI and RSImod. However, this variable strictly evaluates the concentric phase and therefore eliminates the eccentric phase duration of the CMJ and DJ. This ensures comparison of the same variables across the three jumps—albeit produced with different strategies. Further, other variables such as the RSI are not normalized for body mass, whereas the CFI provides a measure of force production and reutilization of the lower limb which is not affected by jump technique and body mass. In this study, it was observed that the CFI (a) varied greatly with no correlation between jumps and (b) did not correlate with JH. This suggests that this novel metric can differentiate between jumps and provide alternative insight into what JH can provide.

### 4.5. Practical Applications

In practice, integrating the CFI with its underlying metrics (rCMF and ConT) enables nuanced athlete monitoring. Practitioners can begin by establishing CFI baselines to create a profile of an athlete’s reliance on contractile elements during the SJ, as well as any SSC shortcomings in the CMJ and DJ. However, the strength of the CFI lies not in one-off testing but in longitudinal monitoring of the CFI and its components over a greater period of time. For example, regular assessments across SJ, CMJ, and DJ can track CFI trends, and divergent improvements (for example, DJ CFI gains without changes in the SJ) could indicate SSC-specific adaptations.

At the cohort level, comparing CFI data across groups can provide normative data and reveal positional trends, such as sport-specific patterns (for example, higher DJ CFI in positions requiring fast SSC like basketball or sprinting versus potential slower SSC emphasis in other sports). This analysis enhances insights into group performance and training periodization, allowing coaches to identify outliers, benchmark against norms, and customize interventions for roles with distinct biomechanical demands.

### 4.6. Limitations

It is important to note that although participants were familiarized with the three jumps prior to testing, this cohort consisted of young males from diverse athletic backgrounds that were not trained jumpers. The cohort homogeneity limits generalizability to other cohorts such as females and elite and older athletes. Investigating other cohorts in order to provide a greater understanding of relationships between the CFI (and its constituents) of each jump and broader physical attributes or athletic capabilities is required.

Further, conducting longitudinal studies to examine how targeted training interventions, specifically aimed at enhancing individual components of the TCM, influence the rCMF and ConT in the SJ, CMJ, and DJ would provide further evidence for this theoretical framework and an alternate paradigm of assessing vertical jump performance.

A further limitation is the use of a single standardized drop height (30 cm) for the DJ, which may not optimize SSC responses for all individuals due to anthropometric and training differences. Future investigations could explore multi-height protocols.

Additionally, inter-individual variations may also stem from unmeasured factors, such as years of jump-specific training, overall experience, or sport specialization. Collecting such data in future studies would refine our understanding.

## 5. Conclusions

In conclusion, there is evidence that the SJ, CMJ, and DJ offer distinct insights into athletic performance. While speculative, these differences may be attributed, in part, to variations in the capabilities of the CC, SEC, and PEC—a theoretical perspective that warrants further empirical investigation to advance our understanding of MTU dynamics. Further, JH in one jump does not uniformly represent an athlete’s performance across all jump types. Consequently, it is recommended that an assessment encompassing the SJ, CMJ, and DJ, along with their respective jump strategy variables, should be employed to offer a comprehensive evaluation of an athlete’s strengths and weaknesses. Such a multifaceted approach to assessment, incorporating multiple metrics, allows for a more nuanced and complete understanding of an athlete’s capabilities. Rather than fixating primarily on JH, it is suggested that assessing the amount of force produced, the duration to generate this force, and accounting for an individual’s mass provides a more comprehensive understanding of athletic performance, particularly when JH is not the sole objective. The CFI, introduced in this study, offers a promising avenue for further investigation.

## Figures and Tables

**Table 1:** Means and standard deviations for the SJ, CMJ, and DJ.

Variable	SJ	CMJ	DJ
JH (cm)	34.6 ± 4.4 _b_	37.8 ± 4.1 _ac_	31.9 ± 5.0 _b_
ConT (ms)	409 ± 48 _bc_	273 ± 24 _ac_	124 ± 15 _ab_
CMF (N)	1263 ± 161 _bc_	1547 ± 202 _ac_	2339 ± 262 _ab_
rCMF (N/kg)	16.2 ± 0.8 _bc_	19.8 ± 1.0 _ac_	30.1 ± 2.7 _ab_
CFI	4.0 ± 0.7 _bc_	7.4 ± 1.0 _ac_	24.9 ± 5.3 _ab_

Note. Abbreviations: CFI = concentric force index; CMF = concentric mean force; ConT = concentric duration; JH = jump height; rCMF = relative concentric mean force. Subscript letters indicate significant differences between jump types (*p* < 0.05)—“_a_” corresponds to squat jump, “_b_” to countermovement jump, and “_c_” to drop jump.

**Table 2:** Correlation between SJ, CMJ, and DJ concentric phase variables.

	Jump Height	Concentric Duration	Concentric Mean Force	Relative Concentric Mean Force	Concentric Force Index
SJ-CMJ	0.691 ‡	0.26	0.901 ‡	0.305	0.303
SJ-DJ	0.098	−0.181	0.65 ‡	0.019	−0.168
CMJ-DJ	−0.47	−0.452 †	0.542 †	−0.223	−0.393

Note. Abbreviations: SJ = squat jump; CMJ = countermovement jump; DJ = drop jump. † Correlation is significant at *p* < 0.05 level and ‡ correlation is significant at *p* < 0.001 level.

**Table 3:** Correlation between Jump height and concentric phase variables.

	Concentric Duration	Concentric Mean Force	Relative Concentric Mean Force	Concentric Force Index
SJ	0.281	−0.041	0.264	−0.103
CMJ	0.198	0.095	0.359	0.002
DJ	0.327	0.212	0.308	−0.035

Note. Abbreviations: SJ = squat jump; CMJ = countermovement jump; DJ = drop jump.

## Data Availability

The data supporting the conclusions of this article will be made available by the authors on request.

## Abbreviations

The following abbreviations are used in this manuscript:

CC	Contractile Component
CFI	Concentric Force Index
CMF	Concentric Mean Force
CMJ	Countermovement Jump
ConT	Concentric Duration
DJ	Drop Jump
GCT	Ground Contact Time
JH	Jump Height
MTU	Muscle-Tendon Unit
PEC	Parallel Elastic Component
rCMF	Relative Concentric Mean Force
RSI	Reactive Strength Index
RSImod	Reactive Strength Index Modified
SEC	Series Elastic Component
SJ	Squat Jump
SSC	Stretch-Shortening Cycle
TCM	Three-Component Model
TCT	Total Contraction Time
vGRF	Vertical Ground Reaction Force
